# Impact of background music on reading comprehension: influence of lyrics language and study habits

**DOI:** 10.3389/fpsyg.2024.1363562

**Published:** 2024-04-05

**Authors:** Yanping Sun, Chuanning Sun, Chang Li, Xinrui Shao, Qingming Liu, Hongen Liu

**Affiliations:** ^1^Department of Applied Psychology, College of Sports and Health, Shandong Sport University, Jinan, China; ^2^School of Physical Education, Shandong University, Jinan, China; ^3^Department of Insurance, Shandong University of Finance and Economics, Jinan, China; ^4^School of Psychology, Qufu Normal University, Qufu, China; ^5^Zizhong Middle School, Linqing, China; ^6^College of Physical Education and Health, Guangxi Normal University, Guilin, China

**Keywords:** reading comprehension, study habits, pop music with lyrics, native language lyrics, second language lyrics, written text language, Chinese college students

## Abstract

Numerous studies have explored the effects of background music on reading comprehension, however, little is known about how native language (L1) lyrics and second language (L2) lyrics in background music influence reading comprehension performance for college students. The present study used a mixed experimental design to examine the effects of listening habits (between-participants variable: non-listeners or listeners), music type (between-participants variable: L1 (Mandarin) pop music, L2 (English) pop music or no music) and text language (within-participants variable: L1 or L2) on reading comprehension of college students in East China. A total of 90 participants (50 females) were screened into non- listeners (*n* = 45) and listeners (*n* = 45), and then were randomly assigned to one of three groups: Mandarin pop music group (*n* = 30), English pop music group (*n* = 30) and no music group (*n* = 30). The results showed that reading comprehension performance was negatively affected by music with lyrics compared to the no music condition. Furthermore, Chinese/English reading comprehension was reduced more by pop music in the same language as the written texts. As expected, non-listeners were more negatively affected by music with lyrics than listeners. For both listeners and non-listeners, average reading comprehension accuracy rates were the lowest in the condition of music with native language lyrics. Overall, our research findings indicate that listening to pop music with lyrics reduces reading comprehension performance. However, listening to background music cause much less distraction if the students commonly listen to music while reading. The current study supports the duplex-mechanism account of auditory distraction.

## 1 Introduction

Listening to music while studying is a common and popular trend for college students. Calderwood et al. (2014) found that 59% of the college students chose to listen to music during a 3-h study session, with 21% listening for more than 90% of the time. Although several studies have demonstrated positive effects of background instrumental music on reading comprehension (Carlson et al., 2004; Khaghaninejad et al., 2016) and second language learning (Kang and Williamson, 2012), irrelevant sound from vocal music may cause auditory distraction from the task at hand (Martin et al., 1988; Furnham and Strbac, 2002; Perham and Currie, 2014; Zhang et al., 2018; Du et al., 2020). Efficient learning is extremely important for college students. However, high levels of auditory distraction will not only affect efficient learning, but also impair mental and physical function and cause irritation and headaches in schools (Astolfi et al., 2019). Thus, it is important to explore the mechanisms that produce auditory distraction. According to the duplex-mechanism account of auditory distraction, the disruptive effect can be induced by *interference-by-process* or *attentional capture* (Marsh et al., 2008, 2009). To date, previous studies investigating the impact of music on reading comprehension have primarily focused on differences between instrumental and lyrical music (e.g., Erten et al., 2015), as well as the influence of differences in musical volume and speed (e.g., Thompson et al., 2012). Notably, these studies have not taken into consideration differences in participant preferences for listening to music while reading. In contrast, the present study investigated how differences in the lyrical language of the same song differentially influence reading comprehension based on reported music-listening habits. With the aim of testing the duplex-mechanism account of auditory distraction, our study explored the interactive effects of native language (L1) lyrics and second language (L2) lyrics in music on reading comprehension performance in L1 and L2 for listeners and non-listeners by using a 3-factor mixed experimental design.

### 1.1 A duplex-mechanism account of auditory distraction

According to the duplex-mechanism account of auditory distraction, there are two functionally different types of auditory distraction. *Interference-by-process* occurs when a similar process used consciously to complete a focal task competes with the involuntary processing of sound. On the other hand, regardless of the task processes involved, attentional capture occurs when the sound triggers a disengagement of attention from the dominant task (Hughes, 2014). For example, semantic speech (e.g., “orange, banana, strawberry”) can cause distraction effects on semantic-based cognitive tasks (e.g., free recall of visually presented words “apple, mango, pear”) (Marsh et al., 2008). According to the interference-by-process theory, semantically similar speech automatically spreads activation through a long-term semantic network, interfering with the similar process of navigating such networks to retrieve information for the focal task (Marsh and Jones, 2010; Hughes, 2014). Interference-by-process explains the semantic distraction effects. Attentional capture falls into two categories: When a sound’s unique content (such as one’s name or one’s native language) gives it the ability to deflect attention, a specific attentional capture takes place. In contrast, when an occurrence draws attention despite having nothing inherently attention-grabbing about it, but rather because of the context in which it takes place, nonspecific attentional capture is created (Eimer et al., 1996). For example, a sound “B” in “CCCCCBCC” or a sound “C” in “BBBBBCBB.” Our study focused on interference-by-process and a specific attentional capture.

### 1.2 The impact of background music on reading comprehension

Reading comprehension, an important and necessary skill for effective academic learning in college, refers to the active process by which individuals understand and construct the meaning of texts based on prior knowledge and experience (Perfetti et al., 2005). Kämpfe et al. (2010) claimed that reading might be more disturbed by vocal music than by instrumental music (Kämpfe et al., 2010). The duplex-mechanism account of auditory distraction has been supported by research evidence demonstrating the disruptive effects of background speech on various memory tasks such as serial short-term memory tasks. However, little is known about supporting evidence from the distraction effects of L1/L2 lyrics on L1/L2 reading comprehension among listeners and non-listeners. According to the simple view of reading model, reading comprehension consists of only two parts, word recognition and language comprehension, and both parts are necessary for reading success (Hoover and Gough, 1990). For college students, mature readers whose word recognition has attained to a level of automation, language comprehension plays the more important role in reading comprehension. Lyrics in music contain semantic information, which will interfere with language comprehension (Martin et al., 1988; Oswald et al., 2000). Thus, we expect that lyrics will induce semantic distraction effects on reading comprehension performance. Our first hypothesis was that the accuracy rates in music conditions would be significantly lower than the accuracy rates with no music for college students (H1).

The impact of background music on reading comprehension is generally contingent on multiple factors such as music types (instrumental or lyric music with various tempos, intensity, familiarity) (Banbury et al., 2001; Hallam and Mac Donald, 2009). In addition to music types, previous studies have confirmed that the effects of music on reading comprehension can be significantly different in various levels of individual diversity (e.g., personality and music preferences) or difficulty of the reading comprehension task (Kiger, 1989; Kallinen, 2002; Anderson and Fuller, 2010). For example, Anderson and Fuller (2010) suggested that disruptive effects of background lyrical music on reading comprehension was more pronounced for 7th- and 8th-grade students exhibiting a stronger preference for the lyrical music, compared with their performance in a quiet environment. Our experimental work focused on identifying interactive effects of music (pop music with L1/L2 lyrics), individual habits (e.g., listening to music in daily study) and tasks (L1/L2 written texts), which helps test whether interference-by-process and a specific attentional capture occurs.

First, pop music is the preferred music genre for most college students (Etaugh and Michals, 1975; Wang and Wang, 2015). For example, Wang and Wang (2015) surveyed 3,688 Chinese college students in Beijing, Inner Mongolia, Shanghai, Henan and Jiangxi regions of Mainland China, and found that: (1) the proportion of college students who liked pop music was as high as 65.05%; (2) 35.23% college students chose “love” as their favorite pop music theme comparing with themes “nostalgic” 33.21%, “witty/humorous” 14.27%, “alternative” 9.49%,“other” 15.73%; (3) 47.85% college student’ favorite singers are from “Hong Kong and Taiwan.” Thus, we choose a masterpiece of classic Mandarin pop music “The Goodbye Kiss” (sung by Jacky Cheung) as the music. Although the song was released in 1993, from its release to 2020, there have been covers of the song by well-known singers almost every year. Specially, this song was covered by Michael Learns to Rock (MLTR) in 2004, and the English version of this song “Take me to your heart” became a classic of international music. Comparing the lyrics of the two songs, the Mandarin lyrics of “The Goodbye Kiss” have a total of 52 sentences, and the whole song is divided into two subsections. The shortest sentence in Mandarin lyrics has a total of five Chinese words, and the longest sentence has 19 words; the English lyrics reproduce the characteristics of the original Chinese sentence well in terms of sentence length and neatness, the shortest sentence consists of four words, and the longest is only 10 words (Wei, 2012). Thus, we chose the pop music with lyrics “The Goodbye Kiss” as our vocal music.

Second, Mandarin Chinese (L1) and English (L2) are the top 2 most spoken languages in the world, and belong to two different language families (Ethnologue, n.d.). Additionally, all Chinese students begin their English study in their third year of primary school or even earlier, and studying English is a key subject for the Chinese college entrance examination required for admission to the university. They will continue to study English to pass College English Test Band 4/6 (CET- 4/6, essential English exams for Chinese college students) in college, and have considerable exposure to English music. English is the most important and widely studied second language for most Chinese college students. Hence, we chose Chinse college students from Mainland China who learn English as a second language for the experiment. Based on the duplex-mechanism account of auditory distraction, when a similar process is used purposefully to accomplish a focal cognitive task and the involuntary processing of sound competes with it, interference-by-process occurs (Hughes, 2014). In our experiment, interference-by-process is produced when lyrics are presented to college students who are deliberately completing a focal reading comprehension task, especially when the lyrics language is the same as the text language in the reading comprehension tasks. That is, the semantic activation of lyrics competes with the semantic access of reading comprehension tasks with the same language as lyrics. Thus, our hypothesis is that Chinese/English reading comprehension accuracy rates when listening to music in the same language would be significantly lower than that in different languages or no music (H2). To be specific, we hypothesized that Chinese reading comprehension accuracy rates when listening to music with Mandarin lyrics would be significantly lower than when listening to music with English lyrics, and English reading comprehension accuracy rates when listening to English music would be significantly lower than when listening to Mandarin music.

Third, students frequently report that listening to music while studying is useful (Etaugh and Ptasnik, 1982), and these students are more likely to form the habit of listening to music in daily study. However, students without the habit instinctively think that music listening can provide a distraction that might affect reading comprehension. Individual differences in inhibitory control may exist between two groups. Inhibitory control refers to the ability to suppress an inappropriate reaction or disregard distracting or irrelevant information, and increased inhibitory control in students probably makes it easier for them to ignore distractions in their surroundings and concentrate on tasks inside and outside of the classroom (Privitera et al., 2022b). However, non-listeners do not develop the habit of listening to music while studying, probably because they have a low level of inhibitory control to concentrate on the focal tasks. Thus, we hypothesized that college students who typically did not report listening to music during study (non-listeners) would have lower reading comprehension accuracy rates than listeners when music was present (H3).

Based on the duplex-mechanism account of auditory distraction, regardless of the quality of target tasks (e.g., Chinese/English comprehension), auditory attentional capture happens whenever a sound produces a disengagement from tasks. Numerous sound varieties (e.g., one’s own name, or her own infant’s screams for a mother) have abilities to specifically captivate attention (Hughes, 2014). Native language (Mandarin Chinese) is familiar and highly dominant, and may cause a specific attentional capture. We expect that both non-listeners and listeners may be more susceptible to auditory distraction when Mandarin music is present rather than English music. That is, in general, people’s ability to understand what they read was worse when they listened to music with native language compared to music in a second language or no music at all. Thus, for both non-listeners and listeners, we hypothesized that average reading comprehension accuracy rates (without distinction between Chinese and English) would be the lowest in the condition of Mandarin music compared with the English/no music condition (H4).

### 1.3 Research questions

In sum, it is worth examining the effects of different habits of listening to music on reading comprehension performance, which can help clarify whether cultivating habits of listening to music while studying is valuable or not. In addition, few studies used both lyrics languages and music-listening habits while study to explore distractive effects of music on reading comprehension. To solve this problem, in this paper, we designed an experiment to explore the effects of music type, written text language and listening habits on reading comprehension among Chinese college students. Our research questions are: (1) would the accuracy rates in music conditions be significantly lower than the accuracy rates with no music for college students? (2) would Chinese/English reading comprehension accuracy rates when listening to music in the same language be significantly lower than that in different languages or no music? (3) would non-listeners have lower L1 and L2 reading comprehension accuracy rates than listeners when music was present? (4) would average reading comprehension accuracy rates (without distinction between Chinese and English) be the lowest in the condition of Mandarin music compared with the English/no music condition?

## 2 Methods

### 2.1 Participants

Before the experiment, we calculated the minimum sample size of each group of participants using G*Power 3.1.9.7 software (Faul et al., 2007) to reach the statistical power. For observing a similar effect to relevant studies (Peng et al., 2017), we use Effect size *f* = 0.22, ɑ = 0.05, 1-β = 0.8 as parameters, number of groups = 6, number of measurements = 2, non-sphericity correction = 1; under the *F* test of ANOVA: repeated measures, within-between interaction (Faul et al., 2021). Hence the total minimum number of participants should be 72, and the minimum number of participants in each large group should be 36.

The participants were screened by filling out a researcher-designed questionnaire of background music listening habits. All participants were recruited randomly from Shandong Sport University in Shandong Province of Mainland China. A total of 90 participants (50 females) between 18 to 21 years of age (Mean = 19.14, SD = 0.92) were selected. Our experiment divided the participants into 2 large groups first: listeners (45 participants) and non-listeners (45 participants). Participants in each large group were randomly assigned to one of three groups: 15 Mandarin pop music group, 15 English pop music group and 15 no music group. All six groups of participants were assigned Chinese and English texts.

Participants were native Mandarin Chinese speakers who started learning English in the third grade of primary school. None of the participants were music majors and English majors, and none of the participants had any formal musical training. They were all right-handed with normal or corrected-to-normal vision. The experimental protocol was approved by the Research Ethics Committee of Shandong Sport University in China, and conducted in compliance with institutional guidelines and regulations. All participants signed an informed consent form prior to the experiment.

### 2.2 Experimental design

This study used a mixed factorial experimental design. There were two between-participants independent variables and a within-participants independent variable. The between-participants variables were listening habits (with two levels: listeners or non-listeners) and music type (with three levels: Mandarin pop music, English pop music or no music). The within-participants variable was text language (with two levels: Chinese or English). The dependent variable was accuracy rates for the reading comprehension tasks. Accuracy rates were defined as the mean percentage of the number of Chinese (English) reading comprehension items answered correctly in the total number of Chinese (English) reading comprehension items.

### 2.3 Materials and apparatus

Materials consisted of a questionnaire, pop music stimuli and written texts. The questionnaire was Researcher-designed Background Music Listening Habits Questionnaire, a self-report survey that was developed to assess participants’ habits of listening to music during study. This scale contained 15 items, each item rated on a Likert 5-point scale ranging from 1 to 5 (1 = Do not agree at all, 2 = Hardly agree, 3 = not sure, 4 = Mostly agree, 5 = Completely agree), and was scored as a continuous variable from 15 (minimum score) to 75 (maximum score). The Cronbach’s ɑ of the scale was 0.87. We used the questionnaire to screen listeners (a total score higher than 60, 60 is the average score of selecting option 4) and non-listeners (a total score lower than 30, 30 is the average score of selecting option 2) to examine distinct effects of listening habits on reading comprehension performance in the formal experiment.

Mandarin song “The Goodbye Kiss” (Mandarin name “Wen3 Bie2,” sung by Jacky Cheung) and English song “Take Me to Your Heart” (sung by Michael Learns to Rock) were used as background music stimuli, as these two songs have the same rhythm and tempo. The two songs were once popular music that are familiar to most Chinese college students. We used a music editor software Adobe Audition CS6 (Adobe Systems Inc., San Jose, CA, United States) to delete the blank space of “The Goodbye Kiss,” and the part with lyrics was kept to ensure that the participants could always be in a music environment with lyrics while carrying out reading comprehension tasks.

Chinese texts (300 character for each text) were selected from simulated tests of the College Entrance Examination; these texts are all about science and technology. English texts (150 words for each text) about education were selected from Public English Test System 3 (PET-3) tests. Preliminary tests were conducted on 120 college students, and finally 7 Chinese texts (coefficient of difficulty between 0.81 and 0.87) and 7 English texts (coefficient of difficulty between 0.85 and 0.90) were selected. There are no significant differences in difficulty coefficient of the 14 written texts. The difficulty coefficient of each text was estimated by the mean number of correct answers/4 (total number of questions). The coefficient of difficulty 0.81 indicates that, on average, three questions were correctly answered by college students. Participants read passages that were two paragraphs long, and then answered four true or false items following each passage. The items include both literal and inferential comprehension questions. Answers to literal questions involve facts such as who, when, where and what, and they can always be found in the texts. For example, “As early as 1909, Max Mow confirmed that there are some cells in the blood that can make blood, True or False.” For inferential questions, participants are required to determine a text’s meaning indirectly by using the information provided in the text. For example, “By the time most students graduate from high school, they spend less time watching TV than they do in class, True or False.” 3 Chinese texts and 3 English texts were used for assessing the levels of reading comprehension of all three groups (L1 pop music, L2 pop music and no music) of participants before the formal experiments. This was done to make sure that there were no significant differences of Chinese and English reading comprehension levels among the three groups. A different set of 3 Chinese texts and 3 English texts were used for the formal experiments. A Chinese text (difficulty coefficient 0.84) and an English text (difficulty coefficient 0.90) were selected for use in the practice phase.

The apparatus consisted of Lenovo laptops (Yoga 14 s, Lenovo Group Ltd., Beijing, China), noise-canceling headphones (SONY WH-1000XM3, Sony Corp., Tokyo, Japan) and E-prime 2.0. The music stimuli, instructions, texts and questions were all presented on Lenovo laptops using programs written in E-prime 2.0 (Psychology Software Tools, Pittsburgh, PA, United States) (Schneider et al., 2012a,b).

### 2.4 Procedure

Participants filled out the informed consent for participating in the study, then were screened by filling out the Questionnaire of Background Music Listening Habits online. Based on the questionnaire total score, the participants were divided into two large group: listeners and non-listeners. Participants in each large group were randomly assigned to one of three groups (Mandarin music, English music and no music). All three groups of participants completed Chinese and English reading comprehension tasks without music before formal experiments, and no significant differences of Chinese and English reading comprehension performance were observed among the three groups.

**Figure 1:**
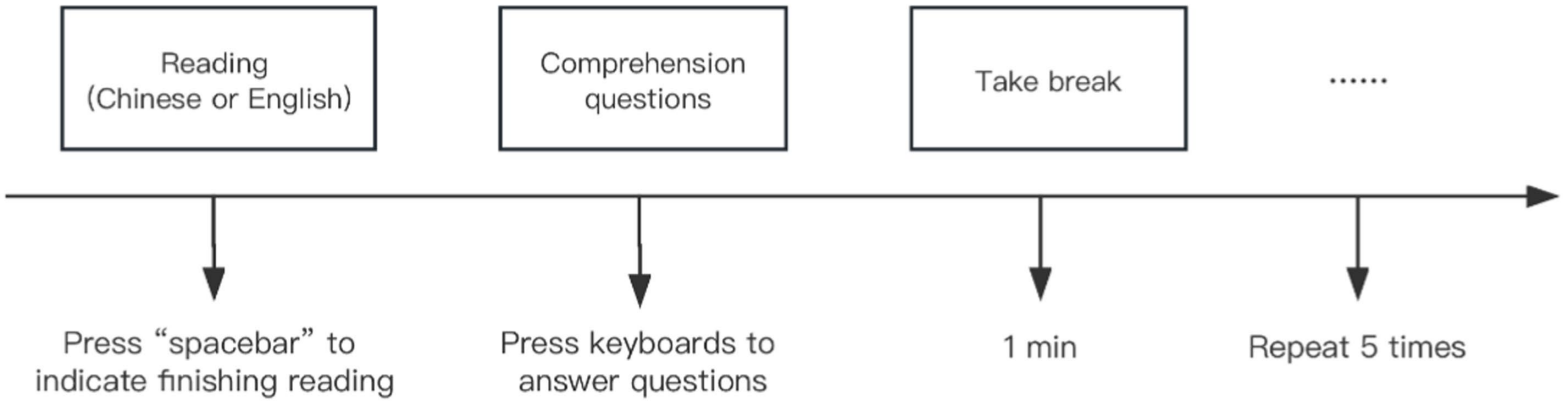
The flow chart of the experimental procedure presented using E-prime 2.0.

In the formal experiment phase, all participants were asked to complete experiment tasks in a quiet lab, with 10 participants in each group seated at individual tables with Lenovo laptops and headphones. First, participants were told to put on headphones and conduct the experiment on Lenovo laptops individually. All music was played between 60 dB ~ 65 dB(A), each participant first put on the headphones and checked to see whether the playback function of the headphones was normal. Then, Participants completed Chinese and English reading comprehension test items under each condition of music type. For each condition, half of the participants read the Chinese text first and the other half read the English text first. The 3 Chinese texts and 3 English texts were presented to participants randomly. After reading each passage, participants pressed the spacebar to end the reading (The maximum reading time for each text is 5 min), and proceeded to answer comprehension questions by pressing “T” (indicating truth) or “F” (indicating false) on keyboards. The flow chart of the experimental procedure presented using E-prime 2.0 was shown in Figure 1.

Participants were asked to answer questions as accurately as possible after reading the passages and to ignore the music. The accuracy rate of each participant was calculated by the total number of Chinese/English items answered correctly/12 (the total number of Chinese/English reading comprehension items). Every participant completed both Chinese texts and English texts in one of three conditions (Mandarin Chinese pop music, English pop music and no music). We tested the effects of listening to music in the same language conditions (L1 music + L1 texts, L2 music + L2 texts) or different language conditions (L1 music + L2 texts, L2 music + L1 texts). For example, participants listening to L1 (Mandarin Chinese) pop music completed L1 (Chinese) texts (the same as lyrics language) and L2 (English) texts (different from lyrics language). Music was played until all participants finished reading comprehension test items.

### 2.5 Statistical analyses

The Statistical Package for the Social Sciences (IBM SPSS, version 23.0; IBM SPSS, Armonk, NY, United States) was used for analysis of the data. The assumptions of ANOVA (homogeneity of variances and normal distribution) were tested. Then the reading comprehension accuracy rates were analyzed using a three-way mixed ANOVA with a within-participants factor (two types of written text language) and two between-participant variables (listening habits and music type). The alpha criterion was set to 0.05. Bonferroni correction was carried out for all *post hoc* analyses.

## 3 Results

One-way ANOVA revealed that baseline reading comprehension performances of three groups (Mandarin music group, English music group and no music group) have no significant difference [Chinese: *F* (2, 87) = 0.226, *p* = 0.718; English: *F* (2, 87) = 0.217, *p* = 0.806].

### 3.1 Descriptive statistics

Means and standard deviations of the reading comprehension accuracy rates are shown in Table 1. A three-way mixed ANOVA for reading comprehension accuracy rates, including two between-participants factors (2 listening habit, 3 music type) and one within-participants factor (2 written text language) was performed (Table 2).

**Table 1:** Reading comprehension accuracy rates [mean (standard deviations)] by group and condition.

Text language	Non-listeners (*n* = 45)			Listeners (*n* = 45)		
	Mandarin music (*n* = 15)	English music (*n* = 15)	No music (*n* = 15)	Mandarin music (*n* = 15)	English music (*n* = 15)	no music (*n* = 15)
Chinese	0.300 (0.070)	0.670 (0.050)	0.900 (0.060)	0.740 (0.070)	0.950 (0.070)	0.900 (0.070)
English	0.500 (0.070)	0.410 (0.070)	0.830 (0.070)	0.920 (0.060)	0.750 (0.060)	0.850 (0.100)

**Table 2:** A three-way analysis of variance (ANOVA) of reading comprehension accuracy rates.

Source	df	*F*	*p*	Partial η^2^*_p_*	Partial η^2^*_p_* 90% CI [LL, UL]
Listening habits	1	634.331	<0.001	0.883	[0.839, 0.901]
Music type	2	232.791	<0.001	0.847	[0.789, 0.871]
Text languages	1	8.362	0.005	0.091	[0.016, 0.195]
Listening habits * music types	2	160.672	<0.001	0.793	[0.723, 0.831]
Music type *text language	2	113.829	<0.001	0.730	[0.642, 0.780]
Listening habits * text language	1	0.339	0.562	0.004	[0.000, 0.053]
Listening habits*music type * texts language	2	1.000	0.372	0.023	[0.000, 0.083]

### 3.2 Main effect analysis and interactive effect analyses

#### 3.2.1 Main effects of music type

We tested our hypothesis (H1) that the accuracy rates in music conditions would be significantly lower than the accuracy rates with no music for college students. We performed a three-way mixed ANOVA for reading comprehension accuracy rates to obtain the main effects and interactive effects. Significant main effects of music type [*F* (2, 87) = 232.791, *p* < 0.001, η^2^*_p_* = 0.847] were observed as shown in Table 2. *Post hoc* analyses revealed the accuracy rates in Mandarin and English music conditions are significantly lower than the accuracy rates with no music (*ps* < 0.01). A mean difference of accuracy rates was −0.081 between Mandarin music and English music condition (95% CI: [−0.110, −0.051]), and was −0.175 between English music and no music condition (95% CI: [−0.205, −0.145]). Thus, the results confirmed our hypothesis H1. The result reveals that music with lyrics decreased reading comprehension performance as compared to no music.

#### 3.2.2 Interactive effects of music type and text language

**Figure 2:**
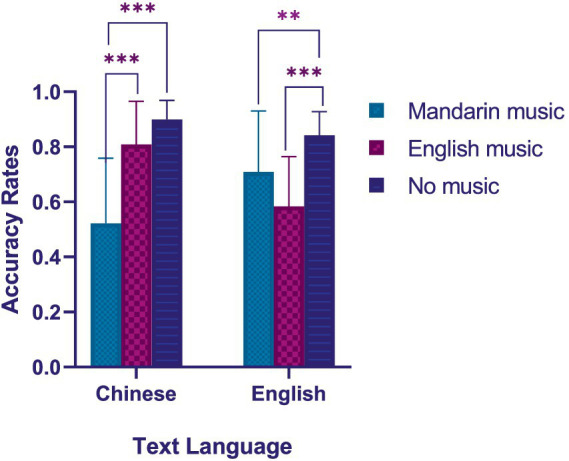
Accuracy rates of Chinese reading comprehension and English reading comprehension for different music types. **p < 0.01; ***p < 0.001.

Our second hypothesis (H2) was confirmed by using a three-way mixed ANOVA. H2 was that Chinese/English reading comprehension accuracy rates when listening to music in the same language would be significantly lower than those with different languages. We observed a significant interaction between music type and text language [*F* (2, 87) = 113.829, *p* < 0.001, η^2^*_p_* = 0.730] as shown in Table 2. For Chinese reading comprehension, as shown in Figure 2, *post hoc* analyses showed that the accuracy rates in Mandarin music group were significantly lower than English music group [*t* (58) = −5.526, *p* < 0.001] and no music group [*t* (58) = −8.420, *p* < 0.001]. A mean difference of Chinese reading accuracy rates was −0.286 between Mandarin music and English music condition (95% CI: [−0.392, −0.180]), and was −0.378 between Mandarin music and no music condition (95% CI: [−0.484, −0.272]). For English reading comprehension, the accuracy rates in the English music group were significantly lower than the Mandarin music group [*t* (58) = −2.385, *p* = 0.023 < 0.05; Figure 2] and the no music group [*t* (58) = −7.041, *p* < 0.001; Figure 2]. A mean difference of English reading accuracy rates was −0.125 between English music and Mandarin music condition (95% CI: [−0.234, −0.016]), and was −0.258 between English music and no music condition (95% CI: [−0.367, −0.150]). These results confirmed our hypothesis H2, and suggested that college students were more distracted by music in the same language as the written texts.

#### 3.2.3 Main effects of listening habits and interactive effects of listening habits and music type

**Figure 3:**
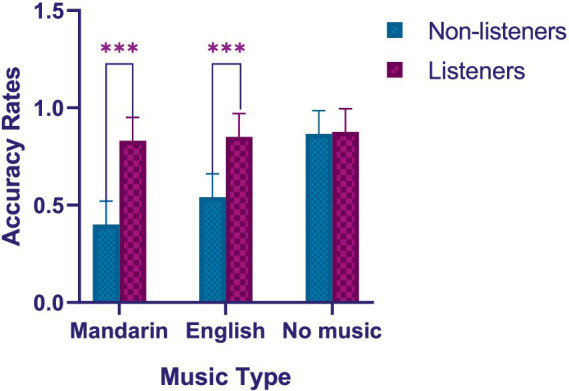
Reading comprehension accuracy rates in different music type groups for different listening habits. ***p < 0.001.

Three-way mixed ANOVA was also used to test our third hypothesis (H3) that non-listeners would have lower reading comprehension accuracy rates than listeners when music was present. The results in Table 2 showed that a significant main effect of listening habits [*F* (1, 88) = 634.331, *p* < 0.001, η^2^*_p_* = 0.883]. *Post hoc* analyses revealed that reading comprehension accuracy rates were lower in non-listeners than listeners (*p* < 0.001). The Table 2 also showed that the interactive effects of listening habits and music type were significant [*F* (2, 87) = 160.672, *p* < 0.001, η^2^*_p_* = 0.793]. *Post hoc* analyses showed significantly lower reading comprehension accuracy rates in the non-listeners compared to listeners, in conditions of music as shown in Figure 3 [Mandarin music: *t* (58) = −138.782, *p* < 0.001; English music: *t* (58) = −99.729, *p* < 0.001]. A mean difference of accuracy rates between non-listeners and listeners was −0.430 in the Mandarin music condition (95% CI: [−0.464, −0.396]), and was −0.309 in the English music condition (95% CI: [−0.343, −0.274]). These results suggest that reading comprehension performance was more negatively affected by music in the non-listeners than in the listeners, confirming our third hypothesis (H3).

**Figure 4:**
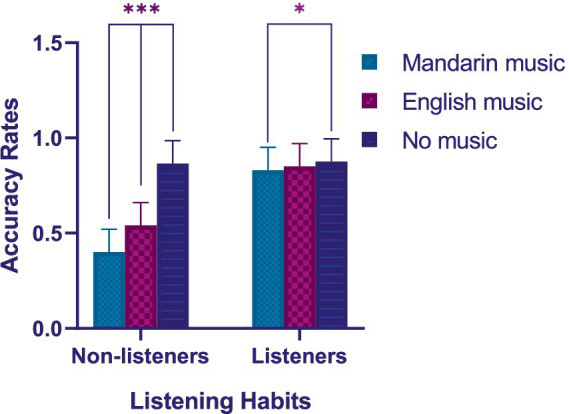
Reading comprehension accuracy rates in different listening habits groups for different music types. *p < 0.05; ***p < 0.001.

Significant interaction effects between listening habits and music type [*F* (2, 87) = 160.672, *p* < 0.001, η^2^*_p_* = 0.793] were observed as shown in Table 2. For the non-listeners, as shown in Figure 4, *post hoc* analyses revealed the accuracy rates while listening to Mandarin music are significantly lower than with English music [*t* (58) = −45.508, *p* < 0.001] and significantly lower than accuracy rates with no music [*t* (58) = −150.401, *p* < 0.001]. A mean difference of reading accuracy rates was −0.142 between Mandarin music and English music condition (95% CI: [−0.183, −0.100]), and was −0.467 between Mandarin music and no music condition (95% CI: [−0.508, −0.425]); For the listener, *post hoc* analyses revealed the accuracy rates while listening to Mandarin music are significantly lower than accuracy rates with no music [*t* (58) = −14.524, *p* < 0.001]. A mean difference of reading accuracy rates was −0.045 between Mandarin music and no music condition (95% CI: [−0.086, −0.003]). Thus, the results also supported our hypothesis H4 that average reading comprehension accuracy rates (without distinction between Chinese and English) would be the lowest in the condition of Mandarin music compared with the English/no music condition for both non-listeners and listeners. These results suggested that music with native language lyrics negatively affected the reading comprehension performance of college students.

## 4 Discussion

The main purpose of this study was to explore the disruptive effects of background music lyrics on first language (L1) and second language (L2) reading comprehension performance among Chinese college students. We also included the influence of music-listening habits by using a 3-factor mixed factorial experimental design. First, our results showed that reading comprehension accuracy rates in music conditions are significantly lower than the accuracy rates with no music. Second, L1/L2 reading comprehension accuracy rates when listening to music in the same language are significantly lower than when listening to a different language. Third, the results showed that significantly lower accuracy rates in non-listeners than listeners when music was played. Finally, for both the non-listeners and listeners, average reading comprehension accuracy rates are the lowest in the condition of Mandarin music compared with English/no music condition. Our results provide experimental evidence in support of distraction effects of L1 or L2 music on L1 and L2 reading comprehension performance among Chinese college students. In addition, our findings also offer additional evidence in favor of the duplex-mechanism account of auditory distraction. Overall, the results support our hypotheses.

### 4.1 The effect of music type

Compared to the no music condition, reading comprehension performance were reduced by music with lyrics. This result is consistent with previous studies which found disruptive effects of vocal music on reading comprehension (Anderson and Fuller, 2010; Perham and Currie, 2014; Ren and Xu, 2019; Dong et al., 2022). Thompson et al. (2012) showed that fast and loud instrumental music disrupts reading comprehension more than slow-tempo music (Thompson et al., 2012). However, though the music in our study is slow-tempo, disruptive effects on reading comprehension were still observed. Lyrics had a significantly detrimental effect on reading comprehension. The finding of the current study supports the *interference-by-process* in the duplex-mechanism account of auditory distraction. According to the interference-by-process, music with lyrics in both L1 and L2 detracted from the performance because semantically processing of the lyrics in these two languages conflicts with semantic processing and access that reading demands (Quan and Kuo, 2023). For comparison, some researchers used musical excerpts in combination with meaningless words as music stimuli. The musical excerpts with meaningless lyrics were unknown to the participants to avoid any associations between the music and semantic or episodic memory. Their results showed neither an enhancing nor a detrimental effect on verbal learning when different styles of background music were played (Jäncke and Sandmann, 2010). However, the present study indicated that music with meaningful lyrics interferes with reading comprehension performance. Language comprehension plays an important role in reading comprehension performance (Hoover and Gough, 1990), and both lyrics and written texts contained semantic information. According to the duplex-mechanism account, from the perspective of the interference-by-process, the semantic interference effects can be explained by assuming that semantic speech triggers automatic spreading of semantic activation over a long-term semantic network that interferes with the analogous process of steering such networks for the purpose of retrieval in the reading comprehension tasks (Marsh and Jones, 2010; Hughes, 2014). Therefore, the lyrics act as competing stimuli with written texts and impair their access to word meaning.

### 4.2 The interaction between music type and text language

Regardless of whether the music and texts were in their L1 or L2 language, Chinese college students were more distracted by music in the same language as the texts. This result indicates that a more detrimental effect on reading comprehension occurred when the auditory input (music lyrics) is the same as the written text language. Based on interference-by-process, the irrelevant semantic information from the speech creates competition for the primary tasks’ dynamic semantic encoding and retrieval processes. As they both vie for semantic access, impairment can therefore be explained in terms of a relative difficulty in choosing the appropriate source of semantic information (Marsh et al., 2009). When lyrics language is the same as the text, the competition process becomes stronger and thus the selection process is more difficult, which causes a more disruptive effect on reading performance. We used music lyrics with L1/L2 as different potential sources of auditory distraction, and the finding provides a further strand of support for interference-by-process.

### 4.3 The effect of listening habits

Our results revealed that reading comprehension performance by the non-listeners were more negatively affected by music than the listeners. These findings are in line with the results of previous studies which showed that people who seldom studied in the presence of background music performed better on reading comprehension tasks in silence (Etaugh and Michals, 1975; Etaugh and Ptasnik, 1982). These results indicate that background music caused detrimental effects for individuals who normally study without music. In contrast, college students who regularly listen to music while studying have much experience of listening to music, and the top-down features (e.g., high working memory and high inhibitory control) can lessen the interference to cognitive activities caused by shared processing of irrelevant information (Quan and Kuo, 2023; Privitera et al., 2023b). Specifically, differences in working memory/inhibitory control between non-listeners and listeners may lead the differential effects of music on reading comprehension, because working memory may generally have an impact on individual ability to carry out cognitive tasks while listening to music (König et al., 2005; Christopher and Shelton, 2017), and it is generally observed that those with high working memory capacity are less easily distracted by irrelevant stimuli (Hughes, 2014). Recent studies also revealed that differences in inhibitory and/or attentional control could predict academic performance including reading (e.g., Privitera et al., 2023b), thus, the relatively low working memory/inhibitory control may make non-listeners were more disrupted by music compared with listeners. In other words, though listeners are negatively affected by music, they are accustomed to reading in the presence of music, thus background music sounds are less distracting for them.

### 4.4 The interaction between listening habits and music type

Our results indicated that for both non-listeners and listeners, music with native language lyrics negatively affected the average reading comprehension performance. The results provide support for the duplex-mechanism account of auditory distraction: in addition to interference-by-process, sound can also produce unnecessary distraction by attentional capture. Music lyrics with the same language as the written texts distract college students by interfering specifically with the similar semantic access processes involved in the reading comprehension task. In contrast, music with native language lyrics disengages students from reading comprehension tasks. Compared to L2 lyrics, native language lyrics are high dominant and more familiar, which may make students rely too much on music rather than keeping them from reading due to music. Thus, a specific attentional capture also caused the auditory distraction. This finding of auditory distraction in different lyrics language conditions provides additional evidence in favor of the duplex-mechanism account.

### 4.5 Limitations and further research

Several limitations should be noted. First, the participants’ English language proficiency, cognitive control and working memory were not assessed. In future study the L2 proficiency can be balanced to explore unique music lyrics effects on reading comprehension, because recent studies have shown that L2 proficiency are correlated to inhibition and attentional control (Privitera et al., 2022a, 2023a), and cognitive control has been found to have a significant impact on academic performance including reading (Privitera et al., 2023b). Working memory/cognitive control can be included as a key variable to explore its effect on reading comprehension while listening to music among non-listeners/listeners. Second, sound without lyrics (e.g., pop music without lyrics or white noise) was not included as one level of music type. Future study can compare reading comprehension performance differences between sound without lyrics group and music with lyrics/no music group to explore the various effects of sound. Third, questions about what music genres participants listen to and their relative frequencies were not included in the researcher-designed questionnaire of background music listening habits. The questionnaire needs to be modified, and should include questions on music genres in future study. Fourth, music type should be manipulated as a within-subject factor instead of a between-subject factor in future study. Finally, this is a behavioral experiment examining music lyrics effects on reading comprehension. With the aim of obtaining the brain and neuroscience evidence to support the duplex-mechanism account of auditory distraction, future studies could explore differences in brain and neural activities when students complete reading comprehension while listening to L1/L2 music, and identify the precise locus of the interference-by-process and attentional capture. These differences may indicate that interference-by-process and attentional capture obtain the functional support of different brain regions which further supports duplex-mechanism account of auditory distraction.

### 4.6 Implications

The current study benefits from several strengths. It is the first study to explore effects of L1 or L2 music lyrics on L1/L2 reading comprehension performance among Chinese college students with different listening habits. For reading comprehension with L1/L2, L1/L2 reading comprehension performance reduced more when the music lyrics language was the same as the written texts. For example, L2 reading performance decreased more when both lyrics and written texts language is L2. In general, for average reading comprehension performance, music with native language lyrics affected it negatively more than L2 music/no music. The current study provided experimental evidence to support the duplex-mechanism account of auditory distraction, and revealed that the duplex-mechanism account can also be applied to auditory distraction of reading comprehension tasks other than serial short-term tasks. The novelty of our study is to distinguish effects of lyrics with native language/s language on L1/L2 reading comprehension. Reading performance difference in lyrics with L1/L2 conditions suggests that auditory distraction has two functionally distinct forms: interference-by-process and attentional capture. The contribution of our research is that choosing music and written texts with L1/L2 helps methodically separate the potential individual contributions of interference-by-process and attentional capture to the overall disruption of task performance.

Our other findings were that reading comprehension performance was reduced by pop music lyrics. In addition, non-listeners were more distracted by lyrics than listeners. These findings have practical implications. Though most college students love pop music, and they usually report that listening to music while studying is beneficial, for college students and educators, it is better not to play pop music with lyrics while students, especially students without music-listening habits, are reading articles whether in their native languages or a second language.

## 5 Conclusion

The present study is an important first step in examining the effects of music with L1 or L2 lyrics on L1/L2 reading comprehension performance among Chinese college students with different listening habits. By using a 3-factor mixed factorial experimental design, we showed that the results verified our hypotheses. Specifically, the key findings are: (1) reading comprehension performance was negatively affected by music with lyrics compared to the no music condition; (2) L1/L2 reading comprehension was more affected by music in the same language as the texts; (3) Non-listeners were more negatively affected by music with lyrics than listeners; (4) For both non-listener and listeners, average reading comprehension accuracy rates are the lowest in the condition of music with native language lyrics. These findings support the claim that college students’ reading performance suffers when they listen to pop music with lyrics compared to no music, and provide experimental evidence support for the duplex-mechanism account of auditory distraction.

## Data availability statement

The raw data supporting the conclusions of this article will be made available by the authors, without undue reservation.

## Ethics statement

The studies involving humans were approved by Research Ethics Committee of Shandong Sport University. The studies were conducted in accordance with the local legislation and institutional requirements. The participants provided their written informed consent to participate in this study.

## Author contributions

YS: Conceptualization, Data curation, Methodology, Supervision, Writing – original draft, Writing – review & editing. CS: Funding acquisition, Writing – original draft, Writing – review & editing. CL: Methodology, Writing – review & editing. XS: Investigation, Writing – review & editing. QL: Investigation, Writing – review & editing. HL: Methodology, Writing – review & editing.

## References

[ref1] Anderson S. A. Fuller G. B. (2010). Effect of music on reading comprehension of junior high school students. Sch. Psychol. 25, 178–187. doi: 10.1037/a0021213

[ref2] Astolfi A. Puglisi G. E. Murgia S. Minelli G. Pellerey F. Prato A.. (2019). Influence of classroom acoustics on noise disturbance and well-being for first graders. Front. Psychol. 10:2736. doi: 10.3389/fpsyg.2019.02736, PMID: 31920797 PMC6923245

[ref3] Banbury S. P. Macken W. J. Tremblay S. Jones D. M. (2001). Auditory distraction and short-term memory: phenomena and practical implications. Hum. Factors 43, 12–29. doi: 10.1518/001872001775992462, PMID: 11474757

[ref4] Calderwood C. Ackerman P. L. Conklin E. M. (2014). What else do college students “do” while studying? An investigation of multitasking. Comput. Educ. 75, 19–29. doi: 10.1016/j.compedu.2014.02.004

[ref5] Carlson J. K. Hoffman J. Gray D. Thompson A. (2004). A musical interlude: using music and relaxation to improve reading performance. Interv. Sch. Clin. 39, 246–250. doi: 10.1177/10534512040390040801

[ref6] Christopher E. A. Shelton J. T. (2017). Individual differences in working memory predict the effect of music on student performance. J. Appl. Res. Mem. Cogn. 6, 167–173. doi: 10.1016/j.jarmac.2017.01.012

[ref7] Dong Y. Zheng H. Y. Wu S. X. Y. Huang F. Y. Peng S. N. Sun S. Y. K.. (2022). The effect of Chinese pop background music on Chinese poetry reading comprehension. Psychol. Music 50, 1544–1565. doi: 10.1177/03057356211062940

[ref8] Du M. Jiang J. Li Z. M. Man D. R. Jiang C. M. (2020). The effects of background music on neural responses during reading comprehension. Sci. Rep. 10:18651. doi: 10.1038/s41598-020-75623-3, PMID: 33122745 PMC7596708

[ref9] Eimer M. Nattkemper D. Schröger E. Prinz W. (1996). “Involuntary attention” in Handbook of perception and action. eds. Neumann O. Sanders A. F., vol. 3 (London: Academic Press), 389–446.

[ref10] Erten O. Ece A. S. Eren A. (2015). The effects of reading with music on reading comprehension. Glob. J. Hum. Soc. Sci. 1, 619–627.

[ref11] Etaugh C. Michals D. (1975). Effects on reading comprehension of preferred music and frequency of studying to music. Percept. Mot. Skills 41, 553–554. doi: 10.2466/pms.1975.41.2.553

[ref12] Etaugh C. Ptasnik P. (1982). Effects of studying to music and post-study relaxation on reading comprehension. Percept. Mot. Skills 55, 141–142. doi: 10.2466/pms.1982.55.1.141

[ref13] Ethnologue. (n.d.). Languages of the World. SIL International. Available at: https://www.ethnologue.com/ (Accessed February 27, 2024).

[ref14] Faul F. Erdfelder E. Lang A.-G. Buchner A. (2007). G*power 3: a flexible statistical power analysis program for the social, behavioral, and biomedical sciences. Behav. Res. Methods 39, 175–191. doi: 10.3758/BF03193146, PMID: 17695343

[ref15] Faul F. Erdfelder E. Lang A.-G. Buchner A. (2021). F test: Fixed effects ANOVA–- special, main effects and interactions. G * Power 3.1 manual. 28–29. Available at: https://www.psychologie.hhu.de/arbeitsgruppen/allgemeine-psychologie-und-arbeitspsychologie/gpower.

[ref16] Furnham A. Strbac L. (2002). Music is as distracting as noise: the differential distraction of background music and noise on the cognitive test performance of introverts and extraverts. Ergonomics 45, 203–217. doi: 10.1080/00140130210121932, PMID: 11964204

[ref17] Hallam S. Mac Donald R. A. R. (2009). “The effects of music in community and educational settings” in The Oxford handbook of music psychology (New York: Oxford University Press), 471–480.

[ref18] Hoover W. A. Gough P. B. (1990). The simple view of reading. Read. Writ. 2, 127–160. doi: 10.1007/BF00401799

[ref19] Hughes R. W. (2014). Auditory distraction: a duplex-mechanism account. PsyCh 3, 30–41. doi: 10.1002/pchj.44 26271638

[ref20] Jäncke L. Sandmann P. (2010). Music listening while you learn: no influence of background music on verbal learning. Behav. Brain Funct. 6:3. doi: 10.1186/1744-9081-6-3, PMID: 20180945 PMC2828975

[ref21] Kallinen K. (2002). Reading news from a pocket computer in a distracting environment: effects of the tempo of background music. Comput. Hum. Behav. 18, 537–551. doi: 10.1016/S0747-5632(02)00005-5

[ref22] Kämpfe J. Sedlmeier P. Renkewitz F. (2010). The impact of background music on adult listeners: a meta-analysis. Psychol. Music 39, 424–448. doi: 10.1177/0305735610376261

[ref23] Kang H. J. Williamson V. J. (2012). The effect of background music on second language learning. In Proceedings of the 12th International Conference on Music Perception and Cognition and the 8th Triennial Conference of the European Society for the Cognitive Sciences of Music, pp. 516–518.

[ref24] Khaghaninejad M. S. Motlagh H. S. Chamacham R. (2016). How does Mozart’s music affect the reading comprehension of Iranian EFL learners of both genders? Int. J. Human. Cult. Stud. 489–499.

[ref25] Kiger D. M. (1989). Effects of music information load on a reading comprehension task. Percept. Mot. Skills 69, 531–534. doi: 10.2466/pms.1989.69.2.531

[ref26] König C. J. Bühner M. Mürling G. (2005). Working memory, fluid intelligence, and attention are predictors of multitasking performance, but polychronicity and extraversion are not. Hum. Perform. 18, 243–266. doi: 10.1207/s15327043hup1803_3

[ref27] Marsh J. E. Hughes R. W. Jones D. M. (2008). Auditory distraction in semantic memory: a process-based approach. J. Mem. Lang. 58, 682–700. doi: 10.1016/j.jml.2007.05.002

[ref28] Marsh J. E. Hughes R. W. Jones D. M. (2009). Interference by process, not content, determines semantic auditory distraction. Cognition 110, 23–38. doi: 10.1016/j.cognition.2008.08.003 19081558

[ref29] Marsh J. E. Jones D. M. (2010). Cross-modal distraction by background speech: what role for meaning? Noise Health 12, 210–216. doi: 10.4103/1463-1741.70499, PMID: 20871175

[ref30] Martin R. C. Wogalter M. S. Forlano J. G. (1988). Reading comprehension in the presence of unattended speech and music. J. Mem. Lang. 27, 382–398. doi: 10.1016/0749-596X(88)90063-0

[ref31] Oswald C. J. P. Tremblay S. Jones D. M. (2000). Disruption of comprehension by the meaning of irrelevant sound. Memory 8, 345–350. doi: 10.1080/09658210050117762, PMID: 11045242

[ref32] Peng S. N. Chen M. J. Wang J. D. (2017). Background music promotes reading comprehension: experimental results with different preferences. J. Jiaying Univ. 10, 96–100.

[ref33] Perfetti C. A. Landi N. Oakhill J. (2005) in The acquisition of Reading comprehension skill, the science of Reading: A handbook. eds. Snowling M. J. Hulme C. (Oxford: Blackwell Publishing), 227–247.

[ref34] Perham N. Currie H. (2014). Does listening to preferred music improve reading comprehension performance? Appl. Cogn. Psychol. 28, 279–284. doi: 10.1002/acp.2994

[ref35] Privitera A. J. Momenian M. Weekes B. S. (2022a). Task-specific bilingual effects in mandarin-English speaking high school students in China. Curr. Res. Behav. Sci. 3:100066. doi: 10.1016/j.crbeha.2022.100066

[ref36] Privitera A. J. Momenian M. Weekes B. S. (2023a). Graded bilingual effects on attentional network function in Chinese high school students. Biling. Lang. Congn. 26, 527–537. doi: 10.1017/S1366728922000803

[ref37] Privitera A. J. Zhou Y. Xie X. (2023b). Inhibitory control as a significant predictor of academic performance in Chinese high schoolers. Child Neuropsychol. 29, 457–473. doi: 10.1080/09297049.2022.2098941, PMID: 35816416

[ref38] Privitera A. J. Zhou Y. Xie X. Huang D. (2022b). Inhibitory control predicts academic performance beyond fluid intelligence and processing speed in English-immersed Chinese high schoolers. Proceedings of the Annual Meeting of the Cognitive Science Society, 44. Available at: https://escholarship.org/uc/item/77r925hr.

[ref39] Quan Y. Kuo Y. L. (2023). The effects of Chinese and English background music on Chinese reading comprehension. Psychol. Music 51, 655–663. doi: 10.1177/03057356221101647

[ref40] Ren Y. N. Xu W. X. (2019). Effect of Chinese and English background music on efficiency on Chinese and English reading comprehension. Adv. Psychol. 9, 978–984. doi: 10.12677/AP.2019.96120

[ref41] Schneider W. Eschman A. Zuccolotto A. (2012a). E-prime User’s guide. Pittsburgh: Psychology Software Tools, Inc.

[ref42] Schneider W. Eschman A. Zuccolotto A. (2012b). E-Prime Reference Guide. Pittsburgh: Psychology Software Tools, Inc.

[ref43] Thompson W. F. Schellenberg E. G. Letnic A. K. (2012). Fast and loud background music disrupts reading comprehension. Psychol. Music 40, 700–708. doi: 10.1177/0305735611400173

[ref9001] Wang L. P. Wang F. (2015). An empirical study on popular songs and the cultivation of college students’ core values. J. Inner Mongolia Norm. Univ. Edu. Sci. 33–37.

[ref44] Wei S. H. (2012). Lyrics Translation under the Guidance of Xu Yuanchong’s Poetry Translation Theory: A Case Study of the English Translation of the Internet Pop Song “The Goodbye Kiss”. Campus English:113+115.

[ref45] Zhang H. Miller K. Cleveland R. Cortina K. (2018). How listening to music affects reading: evidence from eye tracking. J. Exp. Psychol. Learn. Mem. Cogn. 44, 1778–1791. doi: 10.1037/xlm0000544, PMID: 29389184

