# Corrigendum: Impact of background music on reading comprehension: influence of lyrics language and study habits

**DOI:** 10.3389/fpsyg.2024.1464178

**Published:** 2024-07-29

**Authors:** Yanping Sun, Chuanning Sun, Chang Li, Xinrui Shao, Qingming Liu, Hongen Liu

**Affiliations:** ^1^Department of Applied Psychology, College of Sports and Health, Shandong Sport University, Jinan, China; ^2^School of Physical Education, Shandong University, Jinan, China; ^3^Department of Insurance, Shandong University of Finance and Economics, Jinan, China; ^4^School of Psychology, Qufu Normal University, Qufu, China; ^5^Zizhong Middle School, Linqing, China; ^6^College of Physical Education and Health, Guangxi Normal University, Guilin, China

**Keywords:** reading comprehension, study habits, pop music with lyrics, native language lyrics, second language lyrics, written text language, Chinese college students

In the published article, there was an error in the correspondence details. The author Chuanning Sun was erroneously listed as the corresponding author. The correct corresponding author should be Chang Li. The corrected correspondence details appear below.

Correspondence

Chang Li

1803741847@qq.com

The authors apologize for this error and state that this does not change the scientific conclusions of the article in any way. The original article has been updated.

